# Supplementary material for: Trait emotion regulation predicts momentary self-esteem level and variability in adolescents’ daily lives
Source: Commun Psychol. 2025 Oct 31;3:152. doi: 10.1038/s44271-025-00326-2 (PMC12579206; doi:10.1038/s44271-025-00326-2)
Supplement: Supplementary file 2 — Supplementary Information [file 44271_2025_326_MOESM2_ESM.pdf]

Online Supplementary Materials

Supplementary Note 1

Error in the preregistration

In the Analyses Plan section of our preregistration on the OSF (https://osf.io/wdxfe), we stated: “Between-person differences in the within-person residual variance σi^2 (i.e. the individual momentary self-esteem variability) are then modeled by regressing it on adolescents’ self-reported use of ER Strategies and trait self-esteem.”, which could give the wrong impression, that only momentary self-esteem variability was to be regressed on trait self-esteem. In reality, we intended to estimate the more conservative model predicting both level and variability. In line with our initial plan, the Variables section of the preregistration clearly states the approach to insert trait self-esteem as a general control variable. We have now proceeded with this plan.

Supplementary Table 1

Comparison Between Excluded and Included Participants

Variable	*M*		*t*	*p*	Cohen's *d*	95% CI
	Excluded	Included				
Within-person variables						
MSE	5.49	6.28	-2.60	.0110	-0.32	[-0.54, -0.10]
Weekend	0.20	0.29	-1.80	.0765	-0.18	[-0.39, 0.04]
Social interaction partner present	0.61	0.50	2.03	.0451	0.22	[0.00, 0.44]
Between-person variables						
MSE BP	5.30	6.14	-2.29	.026	-0.43	[-0.72, -0.14]
Reappraisal	5.94	5.49	1.36	.178	0.19	[-0.09, 0.48]
Expressive suppression	6.25	5.03	3.57	<.001	0.46	[0.17, 0.75]
Reflection	5.84	6.33	-1.60	.115	-0.21	[-0.50, 0.07]
Social sharing	5.44	6.34	-2.36	.021	-0.32	[-0.61, -0.03]
Age	16.15	16.83	-3.16	.002	-0.48	[-0.77, -0.19]
Gender	0.21	0.18	0.40	.691	0.06	[-0.23, 0.35]
Sample	0.89	0.48	8.16	<.001	0.85	[0.56, 1.14]

Note. Welch *t*-tests. Within-person comparisons are based on 8441 observations and between-person comparisons are based on 461 observations. MSE = Momentary self-esteem. CI = Confidence interval. BP = between-person (momentary variables were averaged across participants and then aggregated to a sample mean). Gender and the sample of original study were dummy coded (0 = female, 1 = male; 0 = Study 1, 1 = Study 2).

Supplementary Table 2

Comparison Between Participants of Study 1 and Study 2

Variable	*M*		*t*	*p*	Cohen's *d*	95% CI
	Study 1	Study 2				
Within-person variables						
MSE	6.50	5.87	10.58	<.001	0.26	[0.21, 0.30]
Weekend	0.29	0.28	0.70	.483	0.02	[-0.03, 0.06]
Social interaction partner present	0.54	0.44	8.81	<.001	0.20	[0.16, 0.25]
Between-person variables						
MSE BP	6.49	5.76	3.88	<.001	0.39	[0.19, 0.58]
Reappraisal	5.82	5.13	3.00	.003	0.30	[0.10, 0.49]
Expressive suppression	4.41	5.71	-4.97	<.001	-0.50	[-0.69, -0.30]
Reflection	6.74	5.88	3.71	<.001	0.37	[0.18, 0.57]
Social sharing	6.84	5.78	3.83	<.001	0.38	[0.19, 0.58]
Age	17.69	15.89	16.37	<.001	1.64	[1.42, 1.87]
Gender	0.24	0.12	3.32	<.001	0.33	[0.13, 0.52]

Note. Welch *t*-tests. Within-person comparisons are based on 8358 observations and between-person comparisons are based on 408 observations. CI = Confidence interval. MSE = Momentary self-esteem. BP = between-person (momentary variables were averaged across participants and then aggregated to a sample mean). Gender and the sample of original study were dummy coded (0 = female, 1 = male; 0 = Study 1, 1 = Study 2).

Supplementary Table 3

Momentary Self-Esteem Regressed on Individual ER Strategies and Control Variables.

	Reappraisal		Expressive Suppression		Reflection		Social Sharing	
	Est.	95% CI	Est.	95% CI	Est.	95% CI	Est.	95% CI
MSE level intercept	**6.23**	[5.94, 6.52]	**6.10**	[5.81, 6.40]	**6.19**	[5.89, 6.50]	**6.16**	[5.85, 6.47]
MSE variability intercept	**0.78**	[0.62, 0.93]	**0.80**	[0.64, 0.95]	**0.78**	[0.63, 0.94]	**0.76**	[0.60, 0.92]
Linear time trend	0.01	[0.00, 0.01]	**0.01**	[0.00, 0.01]	**0.01**	[0.00, 0.01]	**0.01**	[0.00, 0.01]
MSE level								
ER strategy	**0.26**	[0.18, 0.33]	**-0.24**	[-0.30, -0.17]	**0.14**	[0.06, 0.22]	**0.12**	[0.06, 0.19]
Interaction partner present	**0.14**	[0.08, 0.21]	**0.14**	[0.08, 0.21]	**0.14**	[0.07, 0.21]	**0.14**	[0.07, 0.21]
Weekend	**0.06**	[0.01, 0.12]	**0.06**	[0.01, 0.12]	**0.06**	[0.01, 0.12]	**0.06**	[0.01, 0.12]
Age	-0.08	[-0.23, 0.08]	-0.07	[-0.23, 0.08]	-0.06	[-0.22, 0.10]	-0.07	[-0.24, 0.09]
Gender	**0.64**	[0.19, 1.10]	**0.89**	[0.44, 1.35]	**0.85**	[0.37, 1.32]	**0.99**	[0.51, 1.47]
Sample	**-0.58**	[-1.02, -0.14]	-0.41	[-0.85, 0.04]	**-0.59**	[-1.04, -0.12]	**-0.58**	[-1.04, -0.12]
MSE variability								
ER Strategy	-0.04	[-0.08, 0.01]	**0.04**	[0.00, 0.07]	-0.02	[-0.06, 0.03]	0.01	[-0.02, 0.05]
Age	-0.04	[-0.12, 0.05]	-0.04	[-0.12, 0.05]	-0.04	[-0.13, 0.05]	-0.04	[-0.13, 0.05]
Gender	-0.19	[-0.43, 0.06]	-0.23	[-0.47, 0.02]	-0.22	[-0.46, 0.03]	-0.19	[-0.44, 0.06]
Sample	0.12	[-0.13, 0.36]	0.09	[-0.17, 0.33]	0.12	[-0.13, 0.37]	0.15	[-0.10, 0.40]
Residual Variances								
MSE Level	**2.98**	[2.58, 3.48]	**2.94**	[2.54, 3.43]	**3.21**	[2.78, 3.74]	**3.19**	[2.76, 3.72]
MSE Variability	**0.77**	[0.64, 0.92]	**0.77**	[0.64, 0.92]	**0.78**	[0.65, 0.93]	**0.78**	[0.65, 0.93]
Time	**0.00**	[0.00, 0.00]	**0.00**	[0.00, 0.00]	**0.00**	[0.00, 0.00]	**0.00**	[0.00, 0.00]
Explained Variance								
$\Delta R^2_{\text{Level}}$	0.15		0.16		0.09		0.09	
$\Delta R^2_{\text{Variability}}$	0.12		0.13		0.12		0.11	

Note. Results are based on n = 8349 observations nested in 408 individuals. MSE = Momentary self-esteem. Weekend, interaction partner present, gender, and the sample of the original study were dummy coded (0 = weekday, 1 = weekend; 0 = no interaction partner present, 1 = interaction partner present; 0 = female, 1 = male; 0 = Study 1, 1 = Study 2). Estimates reflect unstandardized model results. Estimates concerning momentary

self-esteem variability are on a logarithmic scale. Estimates in bold font have 95% credible intervals not including zero and are considered statistically significant. Time, interaction partner present and weekend were measured at the within-person level, while all remaining predictors were measured at the between-person level. $\Delta R^2_{\text{Level}}$ and $\Delta R^2_{\text{Variability}}$ represent the proportion of variance that can be explained by all model predictors relative to a null model.

Supplementary Table 4

Momentary Self-Esteem Regressed on all ER Strategies.

	Est.	95% CI
MSE level intercept	**6.17**	[5.99, 6.34]
MSE variability intercept	**0.79**	[0.69, 0.88]
Linear time trend	0.00	[0.00, 0.01]
MSE level		
Reappraisal	**0.22**	[0.14, 0.30]
Expressive Suppression	**-0.20**	[-0.28, -0.12]
Reflection	0.01	[-0.07, 0.09]
Social Sharing	-0.01	[-0.08, 0.06]
MSE variability		
Reappraisal	-0.03	[-0.08, 0.01]
Expressive Suppression	**0.06**	[0.02, 0.10]
Reflection	-0.02	[-0.06, 0.03]
Social Sharing	**0.05**	[0.01, 0.09]
Residual Variances		
MSE Level	**2.87**	[2.48, 3.34]
MSE Variability	**0.85**	[0.72, 1.01]
Time	**0.00**	[0.00, 0.00]
Explained Variance		
$\Delta R^2_{\text{Level}}$	0.18	
$\Delta R^2_{\text{Variability}}$	0.03	

Note. Results are based on n = 8349 observations nested in 408 individuals. MSE = Momentary self-esteem. Estimates reflect unstandardized model results. Estimates concerning momentary self-esteem variability are on a logarithmic scale. Estimates in bold font have 95% credible intervals not

including zero and are considered statistically significant. $\Delta R^2_{\text{Level}}$ and $\Delta R^2_{\text{Variability}}$ represent the proportion of variance that can be explained by all model predictors relative to a null model.

Supplementary Table 5

Momentary Self-Esteem Regressed on the Number of ER Strategies Used.

	Absolute dummy coding				Relative dummy coding			
	Without control variables		With control variables		Without control variables		With control variables	
	Est.	95% CI	Est.	95% CI	Est.	95% CI	Est.	95% CI
MSE level intercept	**5.25**	[4.31, 6.20]	**5.55**	[4.60, 6.49]	**5.25**	[4.44, 6.09]	**5.55**	[4.70, 6.37]
MSE variability intercept	**0.59**	[0.08, 1.09]	0.48	[-0.02, 0.98]	**0.71**	[0.26, 1.15]	**0.59**	[0.15, 1.04]
Linear time trend	0.00	[0.00, 0.01]	**0.01**	[0.00, 0.01]	0.00	[0.00, 0.01]	**0.01**	[0.00, 0.01]
MSE level								
Number of used ER strategies	0.27	[0.00, 0.54]	0.21	[-0.06, 0.47]	**0.28**	[0.03, 0.52]	0.22	[-0.02, 0.46]
Interaction partner present	-		**0.14**	[0.08, 0.21]	-		**0.14**	[0.08, 0.21]
Weekend	-		**0.06**	[0.01, 0.12]	-		**0.06**	[0.01, 0.12]
Age	-		-0.06	[-0.23, 0.10]	-		-0.07	[-0.23, 0.10]
Gender	-		**0.80**	[0.32, 1.27]	-		**0.79**	[0.32, 1.26]
Sample	-		**-0.69**	[-1.16, -0.23]	-		**-0.69**	[-1.16, -0.23]
MSE variability								
Number of used ER strategies	0.06	[-0.09, 0.21]	0.09	[-0.05, 0.22]	0.03	[-0.11, 0.16]	0.05	[-0.07, 0.18]
Age	-		-0.04	[-0.13, 0.05]	-		-0.04	[-0.13, 0.05]
Gender	-		-0.22	[-0.46, 0.03]	-		-0.22	[-0.46, 0.03]
Sample	-		0.14	[-0.11, 0.39]	-		0.14	[-0.11, 0.39]
Residual Variances	-				-			
MSE Level	**3.49**	[3.02, 4.06]	**3.30**	[2.85, 3.84]	**3.47**	[3.01, 4.04]	**3.29**	[2.84, 3.83]
MSE Variability	**0.88**	[0.74, 1.04]	**0.77**	[0.65, 0.92]	**0.88**	[0.75, 1.04]	**0.77**	[0.65, 0.93]
Time	**0.00**	[0.00, 0.00]	**0.00**	[0.00, 0.00]	**0.00**	[0.00, 0.00]	**0.00**	[0.00, 0.00]
Explained Variance								
$\Delta R^2_{\text{Level}}$	0.01		0.06		0.01		0.06	
$\Delta R^2_{\text{Variability}}$	0.00		0.12		0.00		0.12	

Note. Results are based on n = 8349 observations nested in 408 individuals. MSE = Momentary self-esteem. Number of used ER strategies represents the sum of emotion regulation strategies classified as used. Each strategy was dummy coded (0 = non-usage, 1 = usage) based on two

criteria: (Absolute dummy coding) an individual mean score ≥ 3 or (Relative dummy coding) an individual mean score within one standard deviation of the sample mean. Weekend, interaction partner present, gender, and the sample of the original study were dummy coded (0 = weekday, 1 = weekend; 0 = no interaction partner present, 1 = interaction partner present; 0 = female, 1 = male; 0 = Study 1, 1 = Study 2). Estimates reflect unstandardized model results. Estimates concerning momentary self-esteem variability are on a logarithmic scale. Estimates in bold font have 95% credible intervals not including zero and are considered statistically significant. Time, interaction partner present and weekend were measured at the within-person level, while all remaining predictors were measured at the between-person level. $\Delta R^2_{\text{Level}}$ and $\Delta R^2_{\text{Variability}}$ represent the proportion of variance that can be explained by all model predictors relative to a null model.

Supplementary Table 6

Momentary Self-Esteem Regressed on Individual ER Strategies and trait self-esteem.

	Reappraisal		Expressive Suppression		Reflection		Social Sharing	
	Est.	95% CI	Est.	95% CI	Est.	95% CI	Est.	95% CI
MSE level intercept	**6.17**	[6.03, 6.31]	**6.17**	[6.03, 6.31]	**6.17**	[6.03, 6.31]	**6.17**	[6.03, 6.31]
MSE variability intercept	**0.79**	[0.69, 0.89]	**0.79**	[0.69, 0.89]	**0.79**	[0.69, 0.89]	**0.79**	[0.69, 0.88]
Linear time trend	0.00	[0.00, 0.01]	0.00	[0.00, 0.01]	0.00	[0.00, 0.01]	0.00	[0.00, 0.01]
MSE level								
ER Strategy	0.04	[-0.02, 0.11]	-0.01	[-0.07, 0.04]	**0.07**	[0.02, 0.13]	0.01	[-0.04, 0.06]
Trait self-esteem	**0.85**	[0.75, 0.94]	**0.86**	[0.77, 0.96]	**0.86**	[0.77, 0.94]	**0.87**	[0.78, 0.96]
MSE variability								
ER Strategy	0.00	[-0.05, 0.04]	0.00	[-0.04, 0.04]	-0.01	[-0.05, 0.03]	**0.04**	[0.00, 0.07]
Trait self-esteem	**-0.16**	[-0.23, -0.10]	**-0.17**	[-0.24, -0.10]	**-0.16**	[-0.23, -0.10]	**-0.18**	[-0.25, -0.12]
Residual Variances								
MSE Level	**1.72**	[1.48, 2.02]	**1.73**	[1.48, 2.02]	**1.70**	[1.46, 1.99]	**1.73**	[1.48, 2.02]
MSE Variability	**0.81**	[0.69, 0.96]	**0.81**	[0.69, 0.96]	**0.81**	[0.69, 0.96]	**0.80**	[0.68, 0.95]
Time	**0.00**	[0.00, 0.00]	**0.00**	[0.00, 0.00]	**0.00**	[0.00, 0.00]	**0.00**	[0.00, 0.00]
Explained Variance								
$\Delta R^2_{\text{Level}}$	0.51		0.51		0.52		0.51	
$\Delta R^2_{\text{Variability}}$	0.08		0.07		0.08		0.08	

Note. Results are based on n = 8349 observations nested in 408 individuals. MSE = Momentary self-esteem. Estimates concerning momentary self-esteem variability are on a logarithmic scale. Estimates reflect unstandardized model results. Estimates in bold font have 95% credible

intervals not including zero and are considered statistically significant. $\Delta R^2_{\text{Level}}$ and $\Delta R^2_{\text{Variability}}$ represent the proportion of variance that can be explained by all model predictors relative to a null model.
