# Supplementary material for: Trait emotion regulation predicts momentary self-esteem level and variability in adolescents’ daily lives
Source: Commun Psychol. 2025 Oct 31;3:152. doi: 10.1038/s44271-025-00326-2 (PMC12579206; doi:10.1038/s44271-025-00326-2)
Supplement: Supplementary file 3 — Reporting Summary [file 44271_2025_326_MOESM3_ESM.pdf]

nature portfolio

Corresponding author(s): DBPR COMMSPSYCHOL-25-0224

Last updated by author(s): Apr 15, 2025

Reporting Summary

Nature Portfolio wishes to improve the reproducibility of the work that we publish. This form provides structure for consistency and transparency in reporting. For further information on Nature Portfolio policies, see our Editorial Policies and the Editorial Policy Checklist.

Statistics

For all statistical analyses, confirm that the following items are present in the figure legend, table legend, main text, or Methods section.

n/a	Confirmed	
☐	☒	The exact sample size (*n*) for each experimental group/condition, given as a discrete number and unit of measurement
☐	☒	A statement on whether measurements were taken from distinct samples or whether the same sample was measured repeatedly
☐	☒	The statistical test(s) used AND whether they are one- or two-sided<br>*Only common tests should be described solely by name; describe more complex techniques in the Methods section.*
☐	☒	A description of all covariates tested
☐	☒	A description of any assumptions or corrections, such as tests of normality and adjustment for multiple comparisons
☐	☒	A full description of the statistical parameters including central tendency (e.g. means) or other basic estimates (e.g. regression coefficient) AND variation (e.g. standard deviation) or associated estimates of uncertainty (e.g. confidence intervals)
☐	☒	For null hypothesis testing, the test statistic (e.g. *F*, *t*, *r*) with confidence intervals, effect sizes, degrees of freedom and *P* value noted<br>*Give P values as exact values whenever suitable.*
☐	☒	For Bayesian analysis, information on the choice of priors and Markov chain Monte Carlo settings
☐	☒	For hierarchical and complex designs, identification of the appropriate level for tests and full reporting of outcomes
☐	☒	Estimates of effect sizes (e.g. Cohen's *d*, Pearson's *r*), indicating how they were calculated

Our web collection on statistics for biologists contains articles on many of the points above.

Software and code

Policy information about availability of computer code

Data collection	The questionnaires were implemented with the open-source software formr.
Data analysis	Data cleaning, preparation and descriptive statistics were conducted in R version 4.4.2 using R Studio version 2024.12.1+563. Statistical models were estimated in Mplus version 8.5. The code of the presented analyses is publicly available on OSF (https://osf.io/9ntck/?view_only=cf8aa5dce0354b3eba057ed8f2fc48bf).

For manuscripts utilizing custom algorithms or software that are central to the research but not yet described in published literature, software must be made available to editors and reviewers. We strongly encourage code deposition in a community repository (e.g. GitHub). See the Nature Portfolio guidelines for submitting code & software for further information.

Data

Policy information about availability of data

All manuscripts must include a data availability statement. This statement should provide the following information, where applicable:

- Accession codes, unique identifiers, or web links for publicly available datasets
- A description of any restrictions on data availability
- For clinical datasets or third party data, please ensure that the statement adheres to our policy

All data used to generate results of the present study are available on OSF (https://osf.io/9ntck/?view_only=cf8aa5dce0354b3eba057ed8f2fc48bf).

Research involving human participants, their data, or biological material

Policy information about studies with human participants or human data. See also policy information about sex, gender (identity/presentation), and sexual orientation and race, ethnicity and racism.

Reporting on sex and gender	Participants self-reported their gender/sex. We did not specify whether responses should reflect gender identity or biological sex, as the German term "Geschlecht" can refer to either. Gender/sex was only considered in the robustness checks as reported in the method and results section.
Reporting on race, ethnicity, or other socially relevant groupings	We did not assess the race or ethnicity of participants because this is uncommon in Germany except for studies focusing on migration, stigmatization, or discrimination. For historical reasons of Nazi dictatorship, it is even seen as potentially unethical and politically incorrect.<br>We assessed age and gender as socio-economic information. Given that all particpants were between 14 and 18 and hat do be enroled into a school, we did not assess any further information. All criteria for selecting the sample can also be found as part of our study Codebooks
Population characteristics	see below: behavioural & social sciences study design
Recruitment	Both studies were disseminated via social media platforms, personal outreach to schools, and leaflets in public spaces.
Ethics oversight	Ethical approval was given by the ethics committee of the German Psychological Society (DGPs; protocol code blinded for review, date of approval: blinded for review) and by the ethics committee of the psychological institute of the University of blinded for review.

Note that full information on the approval of the study protocol must also be provided in the manuscript.

Field-specific reporting

Please select the one below that is the best fit for your research. If you are not sure, read the appropriate sections before making your selection.

☐ Life sciences ☒ Behavioural & social sciences ☐ Ecological, evolutionary & environmental sciences

For a reference copy of the document with all sections, see nature.com/documents/nr-reporting-summary-flat.pdf

Behavioural & social sciences study design

All studies must disclose on these points even when the disclosure is negative.

Study description	The present study is a correlational study. Samples from two experience sampling studies with adolescents were combined for analysis.
Research sample	For this research, we combined data from two original experience sampling studies with adolescent participants. Study 1 (Blinded for review) includes data from students in their final year of high school (Mage = 17.64, SD = 0.93); Study 2 (Blinded for review), includes data from students in different school tracks (Mage = 15.89, SD = 1.22). The final sample comprised 408 adolescents (81.62% female) aged 14 to 22 years (Mage = 16.83, SD = 1.41), who recorded a total of 8,349 ESM entries, with an average of 20.49 ESM entries per person (SD = 9.91, range: 3–35).
Sampling strategy	Convinence sampling was used in this study. Based on a simulation-based power analysis of a prior study that used the same data set and conducted similar types of analyses with different variables (Blinded for review), we expected a satisfactory power well-above .80 to detect small effects with standardized β-coefficients equal to or larger than .10 at an alpha level of .05.
Data collection	The data collection procedure was similar in both studies: Participants first completed several questionnaires during a computer-administered introductory session. They then entered a weeklong ESM period, where participants received five questionnaires per day (9 a.m., 12 p.m., 3 p.m., 6 p.m., and 8 p.m.) on their own smartphones, including questions concerning daily situations, momentary motives, and momentary self-esteem. All questionnaires were implemented with the open-source software formr.
Timing	Study 1 has been assessed at at a university as well as online with the data assessment of T1, which is used here, starting in June 2018 and being finished by the end of December 2019. Study 2 has been assessed completely online. Data assessment of T1, which is used here, started at October 2020 and was finished by the end of December 2020.
Data exclusions	We excluded 53 participants because they reported fewer than three ESM reports, which are necessary to adequately model variability in momentary self-esteem. This procedure was preregistered and differences are reported in Table A1 in the appendix.
Non-participation	We only used data of participants who completed the introductory session. Given the online set-up of both studies, some participants clicked on the initial link but did not provide any actual data. These dropout are reported in our Codebook.
Randomization	Participants were not allocated into experimental groups.

Reporting for specific materials, systems and methods

We require information from authors about some types of materials, experimental systems and methods used in many studies. Here, indicate whether each material, system or method listed is relevant to your study. If you are not sure if a list item applies to your research, read the appropriate section before selecting a response.

Materials & experimental systems

n/a	Involved in the study
☒	☐ Antibodies
☒	☐ Eukaryotic cell lines
☒	☐ Palaeontology and archaeology
☒	☐ Animals and other organisms
☒	☐ Clinical data
☒	☐ Dual use research of concern
☒	☐ Plants

Methods

n/a	Involved in the study
☒	☐ ChIP-seq
☒	☐ Flow cytometry
☒	☐ MRI-based neuroimaging

Plants

Seed stocks	*Report on the source of all seed stocks or other plant material used. If applicable, state the seed stock centre and catalogue number. If plant specimens were collected from the field, describe the collection location, date and sampling procedures.*
Novel plant genotypes	*Describe the methods by which all novel plant genotypes were produced. This includes those generated by transgenic approaches, gene editing, chemical/radiation-based mutagenesis and hybridization. For transgenic lines, describe the transformation method, the number of independent lines analyzed and the generation upon which experiments were performed. For gene-edited lines, describe the editor used, the endogenous sequence targeted for editing, the targeting guide RNA sequence (if applicable) and how the editor was applied.*
Authentication	*Describe any authentication procedures for each seed stock used or novel genotype generated. Describe any experiments used to assess the effect of a mutation and, where applicable, how potential secondary effects (e.g. second site T-DNA insertions, mosiacism, off-target gene editing) were examined.*
